# How Effective and Safe Is Bronchial Thermoplasty in “Real Life” Asthmatics Compared to Those Enrolled in Randomized Clinical Trials?

**DOI:** 10.1155/2016/9132198

**Published:** 2016-09-08

**Authors:** Rita Arrigo, Giuseppe Failla, Nicola Scichilone, Alba La Sala, Carla Galeone, Salvatore Battaglia, Alida Benfante, Nicola Facciolongo

**Affiliations:** ^1^Dipartimento di Biomedicina e Medicina Specialistica, Sezione di Pneumologia, University of Palermo, Palermo, Italy; ^2^Interventional Endoscopic Unit, ARNAS Ospedale Civico Di Cristina Benfratelli, Palermo, Italy; ^3^Pulmonology Unit, Department of Cardiology, Thoracic and Vascular Surgery and Critical Care Medicine, Azienda Ospedaliera ASMN, Istituto di Ricovero e Cura a Carattere Scientifico, Reggio Emilia, Italy

## Abstract

There is limited information on the efficacy and safety of bronchial thermoplasty (BT) in* real life*. We evaluated the outcomes of the randomized clinical trials for BT in severe asthmatics, in whom the exclusion criteria were not strictly controlled. A case series of seven asthmatics (M/F: 4/3; age: 54.6 ± 2.9 years) is reported. Subjects had a statistically significant improvement in AQLQ (from a mean of 3.96 ± 1.1 to 4.5 ± 1.2 and 5.5 ± 0.6 after 6 and 12 months of treatment; *p* = 0.0007) and in the ACQ score (from 2.77 ± 0.8 to 1.83 ± 1.2 and 1.5 ± 0.8 after 6 and 12 months; *p* < 0.001). In the year after BT, severe exacerbations, salbutamol use, and OCS use were significantly lower compared with the 1-yr pretreatment period (*p* < 0.001). No ED visits and hospitalization occurred in the year after BT. No changes in functional parameters were recorded. Our investigation confirms the safety and efficacy of BT in severe asthmatics in* real life* settings.

## 1. Introduction

Bronchial asthma is estimated to affect around 300 million people worldwide, with 5% to 10% of patients suffering from a severe form of the disease that is often refractory to usual treatment [1]. Although inflammation is the underlying mechanism in asthma, alterations of the airway smooth muscle (ASM) contraction represent the hallmark of the disease. ASM contraction is responsible for airway narrowing and airflow obstruction in asthma, and both hypertrophy and hyperplasia of the ASM cells might contribute to the difficulty in obtaining adequate control in some subjects with severe asthma, by causing airflow obstruction.

Treatment of severe asthma is based on the use of high dose of long-acting beta-2 agonists and inhaled corticosteroids (LABA/ICS), possibly associated with other interventions such as oral corticosteroids or anti-IgE treatment. The most recent edition of the GINA guidelines includes bronchial thermoplasty (BT) as a nonpharmacological, selective treatment of severe asthma [2]. BT is a novel intervention that delivers controlled thermal energy to the airway wall through a dedicated catheter during a series of bronchoscopy procedures that result in a prolonged reduction in ASM mass [3, 4], thus ameliorating the symptoms of asthma. Although BT is being increasingly employed in clinical practice, there is limited published information specifically addressing the efficacy and safety of BT in* real life* [5, 6]. We therefore attempted to evaluate the effectiveness of BT in a* real life* sample of individuals suffering from severe asthma undergoing the procedure, by assessing the same primary and secondary outcomes of the main randomized clinical trials for BT.

## 2. Methods

Eligible subjects were adults (18–65 years of age) diagnosed with severe symptomatic asthma according to the GINA guidelines [2], who required regular maintenance medications of inhaled corticosteroids (ICS > 500 mcg/day beclomethasone or equivalent) and a long-acting *β*2-agonist (LABA > 100 mcg/day salmeterol or equivalent). Other medications were allowed, including leukotriene modifiers, omalizumab (if used for at least 1 year), anticholinergic drugs, and oral corticosteroids (OCS). Inclusion criteria were as follows: subjects on stable maintenance asthma medications for at least 4 weeks before entry, prebronchodilator FEV_1_ > 60% of predicted value, and being a nonsmoker for at least 1 year with less than 10 pack-years of smoking history. The only exclusion criteria were the absolute contraindications to the BT: life-threatening asthma, respiratory diseases such as emphysema, use of anticoagulants, and prebronchodilator FEV_1_ < 60% of predicted value.

Subjects were recruited from the Biomedical Department of Internal Medicine and Specialist (DiBiMIS), University of Palermo, Italy, the ARNAS Hospital of Palermo, Italy, and the Pulmonology Unit, Department of Cardiology, Thoracic and Vascular Surgery and Critical Care Medicine, Azienda Ospedaliera ASMN, Reggio Emilia, Italy. All subjects gave their written consent to the procedure and to the use of their clinical information for scientific purposes.

BT was performed by delivering radiofrequency energy to the airway using the Alair Bronchial Thermoplasty System and the Alair Catheter in 3 sessions, about three weeks apart, each lasting about 50 minutes, according to the international recommendations [7]. Subjects were evaluated 4 weeks before the first procedure and at 6 and 12 months after the last procedure. At each visit, physical examination, asthma related quality of life (QoL) by the Asthma Quality of Life Questionnaire (AQLQ), asthma control by the Asthma Control Questionnaire (ACQ), rate of severe exacerbations (i.e., those requiring systemic corticosteroids or doubling of ICS dose), use of asthma medications, and changes in lung function, as well as adverse events, were assessed. Furthermore, emergency department (ED) visits, hospitalizations, days missed from work/school or other activities due to asthma, and rescue medication use were compared to the previous year.

## 3. Results

Seven asthmatics with poorly controlled asthma despite maintenance medical therapy including high dose inhaled corticosteroid (ICS) and long-acting *β*2-agonist (LABA) were included (M/F: 4/3; age: 54.6 ± 2.9 years). Median dose of ICS was 2400 mcg/day beclomethasone or equivalent. Six out of 7 subjects were also under regular oral corticosteroids (OCS, median prednisone or equivalent: 48 mg/day). The functional characteristics of the study subjects are described in Table 1. Subjects showed a statistically significant improvement in AQLQ, which increased from a mean of 3.96 ± 1.1 to 4.5 ± 1.2 after 6 months of treatment and 5.5 ± 0.6 at 12 months (*p* = 0.0007); in particular, 3 subjects reached the minimal clinically important difference of 0.5 at 6 months and 5 subjects reached it at 12 months. All treated subjects showed a statistically significant improvement in asthma symptom control: the ACQ score was 2.77 ± 0.8 before treatment and decreased to 1.83 ± 1.2 after 6 months (*p* = 0.008) and to 1.5 ± 0.8 after 12 months (*p* = 0.001); 4 subjects reached the minimal important difference at 6 months, whereas all subjects achieved it at 12 months. During the posttreatment period, a reduction in number of severe exacerbations compared with pretreatment periods of one year was documented (0.4 ± 0.8/yr versus 4.5 ± 3.6/yr). The OCS dose decreased from 10 ± 8 mg/day of prednisone or equivalent to 8 ± 6 mg/day after 6 months and to 4 ± 4 mg/day after 12 months of treatment (*p* = 0.03). The salbutamol use, in terms of puffs/die, decreased from 1.86 ± 1.7 to 0.4 ± 0.8 after 6 months (*p* = 0.002) and to 0.1 ± 0.4 after 12 months (*p* = 0.008). The absence of ED visits and hospitalization was also documented in the year after BT (from a mean of 3 ± 4.5/yr and 1 ± 2/yr, resp.). The number of days missed from work dropped from 10.6 ± 7.5 in the year before BT to 1 ± 1.8 in the year after BT (*p* = 0.04). As expected, we did not find any significant change in lung functional parameters, such as FEV_1_, VC, FEF_25_, FEF_50_, and FEF_75_ (*p* > 0.05 for all analyses). No serious adverse events were recorded, except for a case of lung atelectasis from fibrin plug, which resolved rapidly [8].

## 4. Discussion

Bronchial thermoplasty is a new FDA-approved treatment of patients with severe asthma; this treatment was developed in order to improve the quality of life and symptom control in patients with severe asthma, consequently reducing the costs associated with the disease. The current observational study aimed to assess the effects of BT in real life, evaluating patients who had already completed the treatment for at least one year. Our observational study replicated in a* real life* context the findings of the Asthma Intervention Research (AIR) 2 clinical trial [9]. This was a randomized, double-blind, sham-controlled trial that enrolled 288 subjects with severe asthma; assessments were completed at 3, 6, 9, and 12 months after the last treatment. Compared with the sham group, the BT group showed a statistically significant improvement in QoL from baseline values (primary outcome). The mean change in integrated AQLQ score was also greater in the BT group (1.35 ± 1.10) than in the sham group (1.16 ± 1.23). During the posttreatment period, a 32% reduction in the rate of severe exacerbations in the BT group compared with the sham group was found. Finally, the BT group reported fewer days lost from work/school or other activities due to asthma (1.32 ± 0.36 d/yr versus 3.92 ± 1.55 d/yr). The current* real life* investigation showed improved quality of life and a reduction in the number of severe exacerbations, ED visits, and working days lost due to asthma, to the same extent of the BT group of AIR 2 study, as well the absence of serious side effects in the posttreatment period. In particular, 79% of our patients reported an improvement of AQLQ score of 0.5 or greater, which is similar to that of the BT group showed in the AIR 2 study (71%). In the year after the BT, the rate of severe exacerbations was 0.4/subject in the* real life* study, compared with 1.2/subject in the BT group of AIR 2 trial; similarly, the number of ED visits was 0/subject versus 0.13, and the number of working days lost due to asthma was 1/subject versus 1.32/subject.

The limits of this study are represented by the small sample of recruited subjects and the potential “placebo” effect due to the active treatment, since no sham group can be designed in* real life* studies. The study is to be intended as a preliminary investigation to demonstrate the safety and efficacy of BT in severe asthmatics in* real life* settings, where inclusion and exclusion criteria may not be strictly controlled. Indeed, our case series presents findings from patients with severe asthma and high number of exacerbations and/or comorbidities who have safely undergone BT in experienced medical centers. To our knowledge, there is only one case series of 8 asthmatics who successfully completed BT despite severe airway obstruction [5] and a study comparing the efficacy outcomes in 10 clinic patients treated with BT and 15 patients enrolled in clinical trials of BT at the same center [6], showing that clinical improvements were more represented in the latter. The present study has the advantage of having included (and treated) subjects whose comorbid conditions would have been exclusion criteria for the clinical trials, such as chronic sinus disease, gastroesophageal reflux, history in the previous year of three or more hospitalizations for asthma, three or more lower respiratory tract infections, and four or more pulses of OCS use for asthma. If anything, the extension of inclusion criteria reinforces the effectiveness of BT. It can also be speculated that although the study did not include an economic evaluation, these findings likely translate into reductions of direct and indirect costs for asthma [10].

In summary, the current* real life* observational study confirms that BT is an effective and safe treatment for severe asthma, even in subjects with high number of exacerbations and/or comorbidities. These findings support the implementation of this procedure in severe symptomatic asthmatics.

## Figures and Tables

**Table 1:** Clinical and lung functional characteristics of the study subjects. Exacerbations, ER visits, and hospital admissions were registered in the previous year.

Subject	Age (yrs)	Gender	Severe exacerbations	ER visits	Hospital admissions	FEV_1_ (% pred)	VC (% pred)	FEV_1_/VC
1	55	F	12	12	1	73	104	0.60
2	35	M	2	0	0	67	66	0.45
3	58	M	2	0	0	78	84	0.70
4	48	F	2	0	0	77	87	0.69
5	69	F	4	6	6	61	110	0.47
6	67	M	6	4	0	80	122	0.63
7	50	M	3	0	0	73	84	0.69
